# Characterization of *gprK* Encoding a Putative Hybrid G-Protein-Coupled Receptor in *Aspergillus fumigatus*

**DOI:** 10.1371/journal.pone.0161312

**Published:** 2016-09-01

**Authors:** Mun-Gu Jung, Sung Su Kim, Jae-Hyuk Yu, Kwang-Soo Shin

**Affiliations:** 1 Department of Life Science, Daejeon University, Daejeon, Republic of Korea; 2 Department of Biomedical Laboratory Science, Daejeon University, Daejeon, Republic of Korea; 3 Departments of Bacteriology and Genetics, University of Wisconsin-Madison, Madison, Wisconsin, 53706, United States of America; Universidade de Sao Paulo, BRAZIL

## Abstract

The G-protein-coupled receptor (GPCR) family represents the largest and most varied collection of membrane embedded proteins that are sensitized by ligand binding and interact with heterotrimeric G proteins. Despite their presumed critical roles in fungal biology, the functions of the GPCR family members in the opportunistic human pathogen *Aspergillus fumigatus* are largely unknown, as only two (GprC and GprD) of the 15 predicted GPCRs have been studied. Here, we characterize the *gprK* gene, which is predicted to encode a hybrid GPCR with both 7-transmembrane and regulator of G-protein signaling (RGS) domains. The deletion of *gprK* causes severely impaired asexual development coupled with reduced expression of key developmental activators. Moreover, Δ*gprK* results in hyper-activation of germination even in the absence of carbon source, and elevated expression and activity of the protein kinase A PkaC1. Furthermore, proliferation of the Δ*gprK* mutant is restricted on the medium when pentose is the sole carbon source, suggesting that GprK may function in external carbon source sensing. Notably, the absence of *gprK* results in reduced tolerance to oxidative stress and significantly lowered mRNA levels of the stress-response associated genes *sakA* and *atfA*. Activities of catalases and SODs are severely decreased in the Δ*gprK* mutant, indicating that GprK may function in proper activation of general stress response. The Δ*gprK* mutant is also defective in gliotoxin (GT) production and slightly less virulent toward the greater wax moth, *Galleria mellonella*. Transcriptomic studies reveal that a majority of transporters are down-regulated by Δ*gprK*. In summary, GprK is necessary for proper development, GT production, and oxidative stress response, and functions in down-regulating the PKA-germination pathway.

## Introduction

Heterotrimeric G protein signal transduction is conserved in all eukaryotes and is crucial for sensing and responding to external signals including nutrients, physicochemical stimuli, and environmental stress. A canonical heterotrimeric G-protein system is composed of a 7-transmembrane (TM) domain G-protein-coupled receptor (GPCR), a heterotrimeric G protein consisting of α, β, and γ subunits, and a down-stream effector [1–4]. GPCRs are sensitized by binding of ligand(s), which changes the GPCR’s interaction with heterotrimeric G proteins. Inactive G proteins are activated via GDP-GTP exchange at the Gα subunit, which results in the dissociation of GTP-Gα from the Gβγ heterodimer. Once dissociated, GTP-Gα, Gβγ, or both can elicit and propagate signals by modulating activities of a number of downstream effectors. Signaling is turned off when GTP is hydrolyzed to GDP by the intrinsic GTPase activity of a Gα subunit, resulting in the reformation of an inactive GDP-Gαβγ hetero-trimer [2, 4]. Key components in modulating G-protein signal transduction are the regulators of G protein signaling (RGS), which assist in quenching the signal by accelerating the intrinsic GTPase activity of the Gα subunit. Signaling can also be turned off by internalization of the GPCR, triggering endocytosis and degradation of the receptor [5].

Based on their potential importance to fungal growth and survival and as potential drug targets, GPCRs have been the subject of numerous bioinformatics studies. As a result, 15 putative GPCRs were identified in *Aspergillus fumigatus*, an opportunistic human pathogenic fungus, which causes allergy and invasive pulmonary aspergillosis (IPA) in immune-compromised patients [6–8]. These GPCRs have been assigned to nine groups based on their phylogenetic relationship with those found in *A*. *nidulans* [6, 9–11]. The two putative pheromone receptors GprA (AFUA3G14330) and GprB (AFUA5G07880) are grouped to Classes I and II, respectively. The Class III GPCR GprC (AFUA7G04800) and GprD (AFUA2G12640), which have high similarity to the *Saccharomyces cerevisiae* Gpr1 [12, 13], might be involved in carbon-source sensing. GprF (AFUA5G04100), GprG (AFUA1G11900), and GprJ (AFUA1G06840) belong to Class IV and might function in nitrogen-sensing, and they are similar to the *Schizosaccharomyces pombe* Stm1 [14]. Class V includes the three putative cAMP receptors GprH (AFUA5G04140), GprI (AFUA3G00780), and GprL (AFUA3G01750), which are similar to the *Dictyostelium discoideum* cAMP receptor cAR1 [6, 10]. GprK (AFUA4G01350) has both a 7-TM and an RGS domain and belongs to Class VI. Class VII includes GprM (AFUA7G05300), similar to rat growth hormone-releasing factor receptors [15]. Class VIII includes GprO (AFUA3G10570) and GprP (AFUA6G07160), similar to yeast Izh zinc regulators [16, 17]. Finally, Class IX is comprised of a single GPCR, NopA (AFUA7g01430), which is similar to bacterial opsins [6].

Previously we characterized functions of GprA, GprB, and GprD in *A*. *nidulans* [9, 18]. The *gprA* and *gprB* genes encode putative GPCRs similar to the yeast pheromone receptors Ste2p and Ste3p, respectively [18]. GprA and GprB are specifically required for self-fertilization. Deletion of *gprA* and/or *gprB* results in formation of reduced numbers of cleistothecia that are smaller than those of wild type and carry few viable ascospores. GprD is involved in the positive regulation of germination and negative control of sexual development in *A*. *nidulans* [9]. Deletion of *gprD* results in delayed conidial germination and enhanced sexual development [9]. In addition, the GprD homologue mediates the increase of intracellular cAMP in response to oxygenated polyunsaturated fatty acids (oxylipins), which act as autocrine and paracrine mediators in human [19].

Only two GPCR-like proteins of *A*. *fumigatus* have been characterized [20]. GprC and GprD that are homologs of Gpr1p in *S*. *cerevisiae* activate the cAMP pathway in response to glucose [13, 21]. Deletion of *A*. *fumigatus gprC* or *gprD* resulted in impaired growth and severely attenuated virulence [20]. In this study, we have investigate the roles of the *gprK* gene in growth, differentiation, nutrient sensing, stress response, and virulence in *A*. *fumigatus*. Results indicate that the putative hybrid GPCR-RGS protein GprK might function as a key upstream controller, governing multiple biological processes in this important human pathogenic fungus.

## Materials and Methods

### Strains, media, and culture conditions

*Aspergillus fumigatus* strains were grown on YPD or MMG and 0.1% yeast extract (YE) at 37°C as previously described [22]. For auxotrophic mutants, the medium was supplemented with 5 mM uridine and 10 mM uracil [23]. For liquid submerged culture, about 5 × 10^5^ conidia/ml were inoculated into liquid MMG with 0.1% YE and incubated at 37°C. For phenotypic analyses of *A*. *fumigatus* strains on air-exposed culture, conidia (1 × 10^6^) of relevant strains were spotted on solid medium and incubated at 37°C for 3 days. To examine development and secondary metabolite production in liquid submerged culture, spores of relevant strains were inoculated to a final concentration of 5 × 10^5^ conidia/ml in 50 ml of liquid MMG with 0.1% YE and incubated at 250 rpm at 37°C for 4 days.

### Generation of the *gprK* null mutant

The oligonucleotides used in the present study are listed in S1 Table. The *gprK* gene was deleted in *A*. *fumigatus* AF293.1 (*pyrG1*) strain [23] by employing double-joint PCR (DJ-PCR) [24]. The deletion construct containing the *A*. *nidulans* selective marker (*AnipyrG*^+^) with the 5' and 3' franking regions of the *gprK* gene was introduced into the recipient strain AF293.1 [25]. The selective marker was amplified from FGSC4 genomic DNA with the primer pair oligo 109/oligo 110. The *gprK* mutant was isolated and confirmed by PCR, followed by restriction enzyme digestion [24]. To complement Δ*gprK*, a single joint PCR (SJ-PCR) method was used [24]. The *gprK* gene’s ORF with presumed promoter and terminator was amplified with specific primer pairs where the 3' reverse primer carries overlapping sequences with the *ptrA* gene’s 5' end. Amplification of the *ptrA* gene was carried out with primer pairs where the 5' forward primer carries overlapping sequences with *gprK* gene’s 3' end. The final amplicon was amplified with the nested primer pair oligo 786/oligo 731 and introduced into a Δ*gprK* strain by transformation. To complement Δ*gprK* with the GPCR domain alone without RGS, the genomic region of *gprK* covering the predicted GPCR region along with the presumed promoter and terminator was amplified with the primer pair (oligo 295/oligo 886). Amplification of the *ptrA* gene was carried out with the primer pairs where the 5' forward primer carries overlapping sequences with the *gprK* gene’s 3' end. The final fusion construct was amplified with the nested primer pair oligo 889/oligo 890 and introduced into a Δ*gprK* strain.

### Nucleic acid isolation and manipulation

To isolate genomic DNA from *A*. *fumigatus*, about 10^6^ conidia were inoculated in 2 ml of liquid MMG + 0.5% YE, and stationary cultured at 37°C for 24 h. The mycelial mat was collected and squeeze-dried, and genomic DNA was isolated as described [24]. The deletion mutant was confirmed by PCR amplification of the coding region of the gene followed by restriction enzyme digestion of the PCR amplicon.

Total RNA isolation was carried out as previously described [9, 26]. Briefly, conidia (5 × 10^5^ conidia/ml) of WT, Δ*gprK* and complement strains were inoculated into liquid MMG with 0.1% YE and incubated at 37°C, 250 rpm. Individual mycelial samples were collected at designated time points from liquid submerged cultures and squeeze-dried. The sample was homogenized using a Mini Bead beater in the presence of 1 ml of TRIzol® reagent (Invitrogen) and 0.3 ml of silica/zirconium beads (Biospec). RNA extraction was performed according to the manufacturer’s instruction (Invitrogen). Quantitative RT-PCR (qRT-PCR) assays were performed according to the manufacturer's instruction (Qiagen, USA) using 96-well optical plates and a Rotor-Gene Q (Qiagen, USA). Each run was assayed in triplicate in a total volume of 20 μl containing the RNA template, One Step RT-PCR SYBR Mix (Doctor Protein, Korea), reverse transcriptase, and 10 pmole of each primer (S1 Table). Reverse transcription was performed at 42°C for 30 min. PCR conditions were 95°C/5 min for one cycle, followed by 95°C/30 s and 55°C/30 s for 40 cycles. Amplification of one single specific target DNA was checked by melting curve analysis (+0.5°C ramping for 10 s, from 55°C to 95°C). The expression ratios were normalized to EF1α expression and calculated according to the ΔΔCt method [27].

### Phenotypic analyses

Germination rates were measured as previously described with a slight modification [28]. To examine germination levels, conidia of WT and mutant were inoculated in 5 ml of liquid MMG with 0.1% YE, or liquid medium lacking a carbon source, and incubated at 37°C. Levels of germination were examined every 2 h after inoculation under a microscope.

To assess the effects of a variety of carbon sources (1%), strains were grown on a variety of media, the base being MMG as described above. About 1×10^6^ spores of each strain in 0.1% Tween 20 were inoculated and incubated at 37°C for 3 days with three replicate plates per condition.

Various media were used to assess the roles of GprK in stress responses. For oxidative stress test, hydrogen peroxide (10 mM), menadione (100 μM), and paraquat (100 μM) were added to the YG media after autoclaving and cool down. The following stressors were added to the YG media after autoclaving to assess cell wall stress and osmotic stress: Congo red (100 μg/ml), calcofluor white (100 μg/ml), caspofungin (0.2 μg/ml), sodium chloride (1 M), and sorbitol (1 M). To assess pH stress, media were buffered to three different pH levels (4.0, 6.0, and 8.0) before autoclaving.

To assess the production of gliotoxin (GT), conidia of each strain were inoculated into 50 ml liquid MMY and incubated for 7 days at 37°C and 280 rpm. GT was extracted with chloroform as described previously [29]. The chloroform extracts were air-dried and resuspended in 100 ml of methanol. Ten μl aliquots of each sample were applied to a thin-layer chromatography (TLC) silica plate containing a fluorescence indicator (Kiesel gel 60, E. Merck). The TLC plate was developed with toluene:ethyl acetae:formic acid (5:4:1, v/v) until the solvent front reached about 15 cm. GT standard was purchased from Sigma (USA).

### PKA, SOD, and catalase assay

*A*. *fumigatus* strains were grown in MMG with 0.1% YE for 24 h at 37°C. Mycelia were suspended in the lysis buffer (50 mM Tris-HCl pH 7.2, 150 mM NaCl, 1% Triton X-100, 1 mM EDTA) and homogenized using a Mini Bead-Beater (BioSpec Products). The homogenate was centrifuged in a microcentrifuge for 5 min at 15,000 rpm at 4°C, and the supernatant (10 μl, 3 mg/ml) was used in assay for PKA activity using fluorescent dye-coupled kemptide peptide (Promega, USA) as the photoacceptor according to the manufacturer's instructions. For catalase and SOD activity assays, protein was extracted from the conidia of WT, mutant, and complement strains. Conidia of each strain were suspended in the lysis buffer and homogenized using a Mini Bead-Beater (BioSpec Products). The homogenate was centrifuged in a microcentrifuge for 5 min at 15,000 rpm at 4°C, and the supernatant was used for further analyses. For the detection of catalase activity on gels, the mycelial extracts were subjected to non-denaturing PAGE, and the ferricyanide-negative stain was used to locate bands containing catalase activity [30]. SOD activity on a gel was visualized by inhibition of the reduction of NBT (Sigma) according to the method of Beauchamp and Fridovich [31].

### Virulence assay

Conidia invasion assay was performed with the type II human alveolar cell line A549. A549 cells were seeded in 24-well plates at a concentration of 1 × 10^5^/well and incubated at 37°C for 1 h. Conidia suspension (100 μl) was added to the cells at a multiplicity of infection (MOI) of 10 and incubated for 4 h at 37°C. The cells were then washed five times with PBS, and Triton X-100 (200 μl/well) was added to the well in order to disrupt the cells and release the intracellular conidia. After harvesting the conidia by centrifugation, the conidia were resuspended in 600 μl PBS and 100 μl aliquots of the resuspended conidia were incubated on MMG at 37°C for 36 h.

The insect survival assay was performed as previously described with some modifications [32]. Briefly, sixth instar *G*. *mellonella* used for experiments were selected to be similar in size (approximately 0.3 g). Larvae were infected by injecting the fresh conidia (1 × 10^5^, 5 μl) into the last left pro-leg. The petri dishes were stored in a container loosely covered with aluminum foil and incubated at 37°C in the dark for the duration of the experiment. Larvae were checked daily for survival and larvae that did not respond to stimulation were recorded as dead. The larvae were fixed by immersion in 10% (v/v) neutral buffered formalin for 3–7 days. The larvae were blocked by a longitudinal section and sections were embedded in paraffin wax. Thin sections of the paraffin-embedded tissue were then stained with hematoxylin and eosin (H&E) and Periodic acid-Schiff (PAS) for microscopic examination.

### Microarray analysis

For control and test RNAs, the synthesis of target cDNA probes and hybridization were performed using Agilent’s Low Input Quick Amp WT Labeling Kit (Agilent Technology, USA) according to the manufacturer’s instructions. Briefly, 100 ng total RNA was mixed with WT primer mix and incubated at 65°C for 10 min. cDNA master mix (5× First strand buffer, 0.1 M DTT, 10 mM dNTP mix, RNase-Out, and MMLV-RT) was prepared and added to the reaction mixture. The samples were incubated at 40°C for 2 h, and then the RT and dsDNA synthesis reactions were terminated by incubating at 70°C for 10 min. The transcription master mix was prepared as directed by the manufacturer’s protocol (4× Transcription buffer, 0.1 M DTT, NTP mix, 50% PEG, RNase-Out, inorganic pyrophosphatase, T7-RNA polymerase, and Cyanine 3/5-CTP). Transcription of dsDNA was performed by adding the transcription master mix to the dsDNA reaction samples and incubating at 40°C for 2 h.

Amplified and labeled cRNA was purified on an RNase mini column (Qiagen) according to the manufacturer’s protocol. Labeled cRNA target was quantified using a ND-1000 spectrophotometer (NanoDrop Technologies, USA). After checking labeling efficiency, each of cyanine 3-labeled and cyanine 5-labeled cRNA target were mixed, and fragmentation of cRNA was performed by adding 10× blocking agent and 25× fragmentation buffer and incubating at 60°C for 30 min. The fragmented cRNA was resuspended with 2× hybridization buffer and directly pipetted onto assembled MYcroarray.com (*A*. *fumigatus* AF293) 30K Microarray. The arrays hybridized at 57°C for 17 h using an Agilent Hybridization oven (Agilent Technology, USA). The hybridized microarrays were washed as per the manufacturer’s washing protocol (Agilent Technology, USA). Hybridization images were analyzed by an Agilent DNA microarray Scanner (Agilent Technology, USA), and the data quantification was performed using Agilent Feature Extraction software 10.7 (Agilent Technology, USA). The average fluorescence intensity for each spot was calculated and local background was subtracted using Gene Pix Pro 6.0 (Axon Instruments, USA). Loess normalization and selection of fold-changed genes were performed using GenoWiz 4.0 (Ocimum biosolutions, India). The data is available in the Gene Expression Omnibus (GEO) at NCBI (the accession number = GSE83200).

## Results

### Summary of *A*. *fumigatus* GprK

Based on their domain structures, GprK was aligned and compared with other putative GPCRs in *A*. *fumigatus* (S1A Fig). GprK consists of 559 amino acids and contains a 7-TM domain, 2 low compositional complexity regions, and an RGS domain at the C-terminal end. This RGS domain consists of 174 amino acids and starts at position 372 and ends at position 545 (E-value; 1.9e-8). This GprK and its potential homologs in *Neosartorya fischeri* (*A*. *fischerianus*), *A*. *clavatus*, *A*. *niger*, *A*. *oryzae*, *A*. *flavus*, *A*. *terreus*, and *A*. *nidulans* were aligned and a phylogenetic tree was generated (S1B Fig). GprK of *A*. *fumigatus* shares 47 and 62.5% identity with the hypothetical protein of *A*. *niger* CBS 513.88 (An04g07760) and GprK of *A*. *nidulans* FGSC A4 (AN7795), respectively (S1C Fig).

### GprK is required for proper asexual development

**Fig 1:**
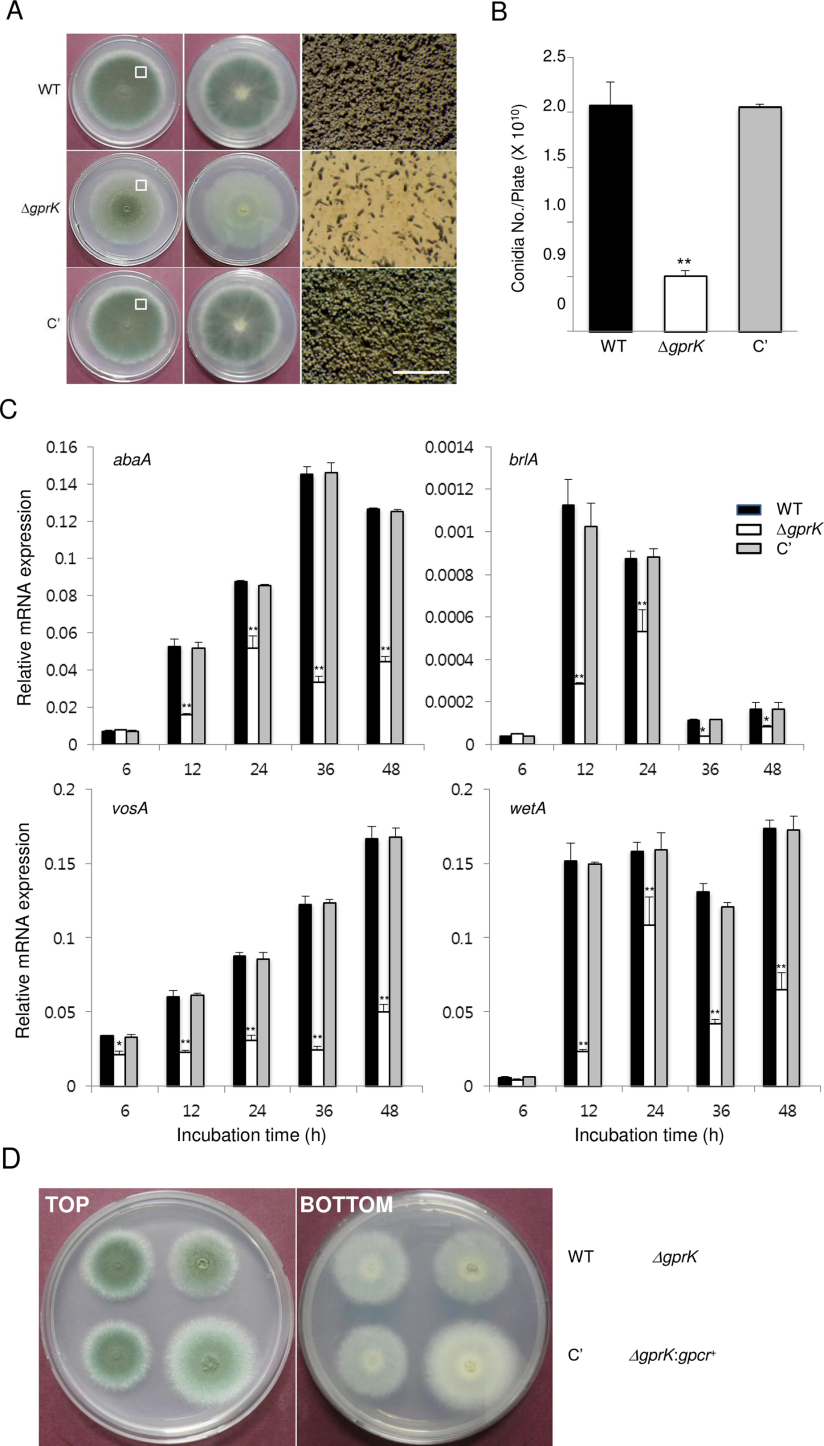
The role of GprK in asexual development. (A) Colony photographs of WT (AF293), Δ*gprK*, and complemented (C') strain point-inoculated on solid MMG with 0.1% YE and grown for 3 days (Top: left; Bottom: middle panels). The enlarged photographs from the plate (indicated by the white box) are shown in the right panels with the bar indicating 1 mm. (B) Conidia numbers produced by each strain per plate. Student’s *t*-test: *p < 0.05, **p < 0.01. (C) mRNA levels of the asexual developmental regulator genes in WT, Δ*gprK*, and C' strains determined by quantitative PCR (qRT-PCR). Cultures were incubated in liquid MMY and mRNA levels were normalized using the *ef1α* gene, according to the ΔΔCt method. Data are expressed as the mean ± standard deviation from three independent experiments. Student’s *t*-test: *p < 0.05, **p < 0.01. (D) Photographs of colonies of WT, Δ*gprK*, C', and Δ*gprK*:*gpcr*^+^ strains point-inoculated on solid MMG and grown for 3 days (Top: left; Bottom: right panels).

To characterize functions of GprK in asexual development, we generated the Δ*gprK* mutant by replacing the ORF with the *A*. *nidulans pyrG* marker. We also generated complemented strains (C') via re-introducing the wild type (WT) allele of *gprK* to a deletion strain. As shown in Fig 1A, the radial growth rate of the Δ*gprK* mutant colony on solid medium was similar to those of WT and C' strains. Although the *gprK* deletion strain demonstrated no change in radial growth, it formed very faint colonies with highly reduced thallic density and significantly lowered formation of conidiophores compared to WT and C’ strains (Fig 1A). Moreover, whereas the colony edge of WT and C' strains contained an abundance of conidiophores, the Δ*gprK* mutant exhibited a relatively small number of conidiophores (Fig 1A, right panels). Quantitative analyses of conidia per plate grown on solid medium further demonstrated that asexual spore production in the Δ*gprK* mutant (0.50 × 10^10^ conidia/plate) was dramatically decreased to a level that was only about 25% of WT and C' strains (Fig 1B). We then analyzed mRNA levels of key asexual developmental regulators, *abaA*, *brlA*, *vosA*, and *wetA*, and found that the deletion of *gprK* resulted in significantly reduced levels of their mRNA at all times tested (Fig 1C). These results suggest that GprK is necessary for both proper control of conidiation on air-exposed solid culture and for developmental regulator expression. To check whether the GPCR domain alone in GprK is sufficient to restore the growth and developmental phenotypes, we generated five individual strains with the GPCR domain alone in the *gprK* null background (Δ*gprK*:*gpcr*^+^) and analyzed their phenotypes. As shown in Fig 1D, while Δ*gprK*:*gpcr*^+^ strains exhibited the faint and low-thallic density phenotype like the Δ*gprK* mutant, they all showed enhanced radial growth compared to WT, C’, and mutant strains. These results indicate that both GPCR and RGS domains are necessary for the full functionality of GprK, and its GPCR domain without the RGS domain may alter the control of vegetative growth.

### GprK downregulates spore germination and cAMP signaling pathway

**Fig 2:**
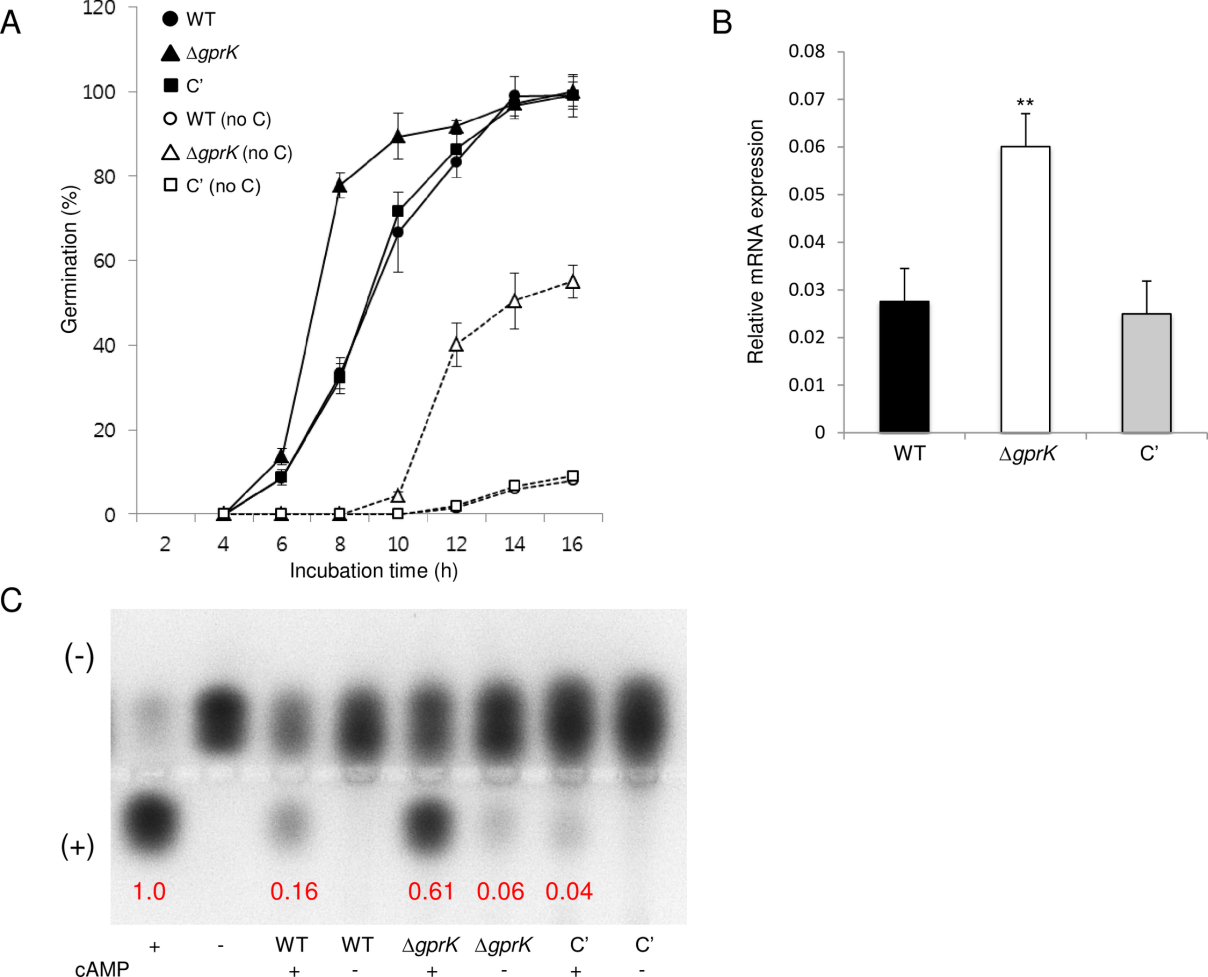
The role of GprK in spore germination and PKA activity. (A) Kinetics of germ outgrowth in *A*. *fumigatus* strains when inoculated in liquid MMG at 37°C in the presence or absence (“no C”) of glucose. (B) Accumulation of *pkaC1* mRNA in WT, Δ*gprK*, and C' strains analyzed by qRT-PCR. Student’s *t*-test: **p < 0.01. (C) PKA activity of *A*. *fumigatus* strains as monitored by gel electrophoresis. A phosphorylated substrate migrates toward the cathode (+). Each strain was grown in MMG for 24 h at 37°C, at which time a mycelial extract was analyzed. Note that the expression of *pkaC1* mRNA and PKA activity were significantly increased in the Δ*gprK* mutant strain compared to WT and C' strains.

To investigate a potential role of GprK in controlling spore germination, we analyzed the kinetics of germ tube emergence in the Δ*gprK* mutant in comparison to that of WT and C' strains. In the presence of glucose, conidia of all strains began to germinate at 6 h of incubation. However, at 8 h, while ~35% of WT and C' strains conidia germinated, 80% of the Δ*gprK* mutant conidia germinated, suggesting that GprK may negatively regulate conidial germination. This was more evident in the absence of carbon source, where the germination time and rate of Δ*gprK* strain conidia germination were more rapid and higher than those of WT and C' strains (Fig 2A).

Previous studies have demonstrated that the heterotrimeric G-protein composed of GanB, SfaD, and GpgA activates a cAMP-dependent protein kinase (PKA) pathway in response to glucose, whose signaling is attenuated by RgsA in *A*. *nidulans* [33–35]. In *A*. *nidulans*, PkaA is the primary PKA that positively functions in vegetative growth and spore germination but negatively controls asexual sporulation [28]. As the deletion of *gprK* resulted in hyper-active germination and reduced conidiation in *A*. *fumigatus*, we tested whether this deletion altered levels of PkaC1, a major catalytic subunit of PKA in *A*. *fumigatus*. As shown in Fig 2B, *pkaC1* mRNA levels were about two-fold higher in the Δ*gprK* mutant than in WT and C' strains. PKA activity was assessed using colored kemptide, a peptide substrate specifically recognized and phosphorylated by PKA. As shown in Fig 2C, WT and C' strains exhibited very little PKA activity until cAMP was added. In contrast, PKA activity in the Δ*gprK* strain was clearly detectable even in the absence of cAMP, and the activity increased about 10-fold in the presence of cAMP. These results indicate that GprK may negatively control cAMP-dependent signaling pathway, which could explain the apparent developmental and germination phenotypes of the Δ*gprK* mutant.

### GprK functions in carbon source sensing and oxidative stress response

**Fig 3:**
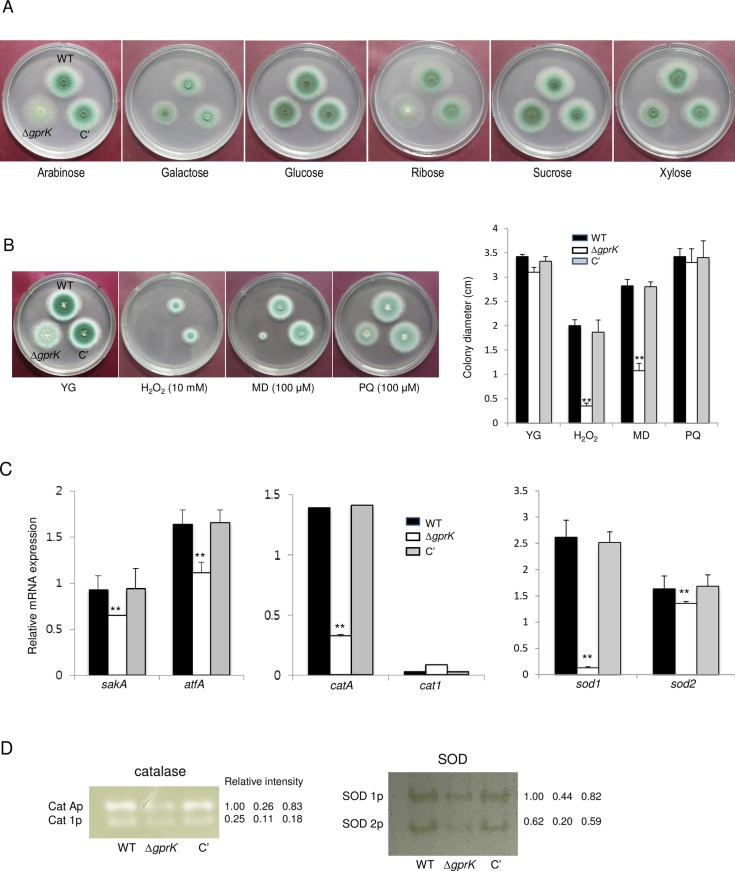
Nutrient sensing and stress responses mediated by GprK. (A) Effect of various carbon sources on the growth of WT, Δ*gprK*, and C' strains. Note that the growth of the mutant was severely restricted on arabinose, ribose, and xylose compared to WT and C' strains. (B) Radial growth of WT, Δ*gprK*, and C' strains in presence of oxidative stressors H_2_O_2_, menadione (MD), or paraquat (PQ) at indicated concentrations following incubation at 37°C for 48 h. (C) Levels of oxidative stress-related genes’ mRNA in WT, Δ*gprK*, and C' strains analyzed by qRT-PCR. Statistical significance was determined by a Student’s *t*-test: *p < 0.05 and **p < 0.01. (D) Conidial catalases and SODs activities of WT, Δ*gprK*, and C' strains shown in non-denaturing polyacrylamide gels.

The presence or absence of carbon and nitrogen sources can be detected by various fungal GPCRs, including Gpr1 of *S*. *cerevisiae* [12, 13, 36] and GPR-4 of *Neurospora crassa* [37]. However, a nutrient-sensing GPCR has not been identified in the aspergilli except in *A*. *flavus* [38]. The Δ*gprA*, Δ*gprC*, Δ*gprJ*, Δ*gprK*, and Δ*gprR* mutants of *A*. *flavus* were growth impaired on several carbon sources [38]. To investigate a potential role for the *A*. *fumigatus* GprK in carbon source sensing, the Δ*gprK* strain was grown on a variety of carbon sources such as arabinose, galactose, glucose, ribose, sucrose, and xylose. As shown in Fig 3A, while there were no differences in growth on hexoses, the Δ*gprK* mutant was severely growth restricted with arabinose or ribose as the carbon source, suggesting that GprK may play a role in pentose sensing.

We then asked whether GprK is associated with stress responses by exposing the Δ*gprK* mutant to a variety of stressors and measuring the radial growth. Distinct from the Δ*gprK* mutant of *A*. *flavus*, which was sensitive to Congo red (200 μg/ml), 1 M sodium chloride, and acidic pH [38], the Δ*gprK* mutant of *A*. *fumigatus* did not exhibit altered tolerance to cell wall stressors, osmotic stressors, and pH stress (S2 Fig). However, the *A*. *fumigatus* Δ*gprK* mutant was highly sensitive to various oxidative stressors including hydrogen peroxide and menadione (MD) but not paraquat (PQ) (Fig 3B). Reductions of approximately 82 and 61% in colony radial growth for the Δ*gprK* mutant were observed in the presence of hydrogen peroxide and MD, respectively. To determine a potential contribution of GprK in a MAPK pathway, we analyzed mRNA levels of stress-response related genes in addition to activity of catalases and superoxide dismutases (SODs) in conidia. The expression of *sakA* and *atfA* mRNA was significantly reduced in Δ*gprK* mutant conidia, and accumulation of catalase and SOD mRNAs were lowered in Δ*gprK* strain (Fig 3C). The activities of CatA, SOD1, and SOD2 were drastically reduced in Δ*gprK* strain (Fig 3D), too. These results indicate that GprK is required for proper resistance of the fungus to certain oxidative stresses.

### A role of GprK in gliotoxin production and virulence

**Fig 4:**
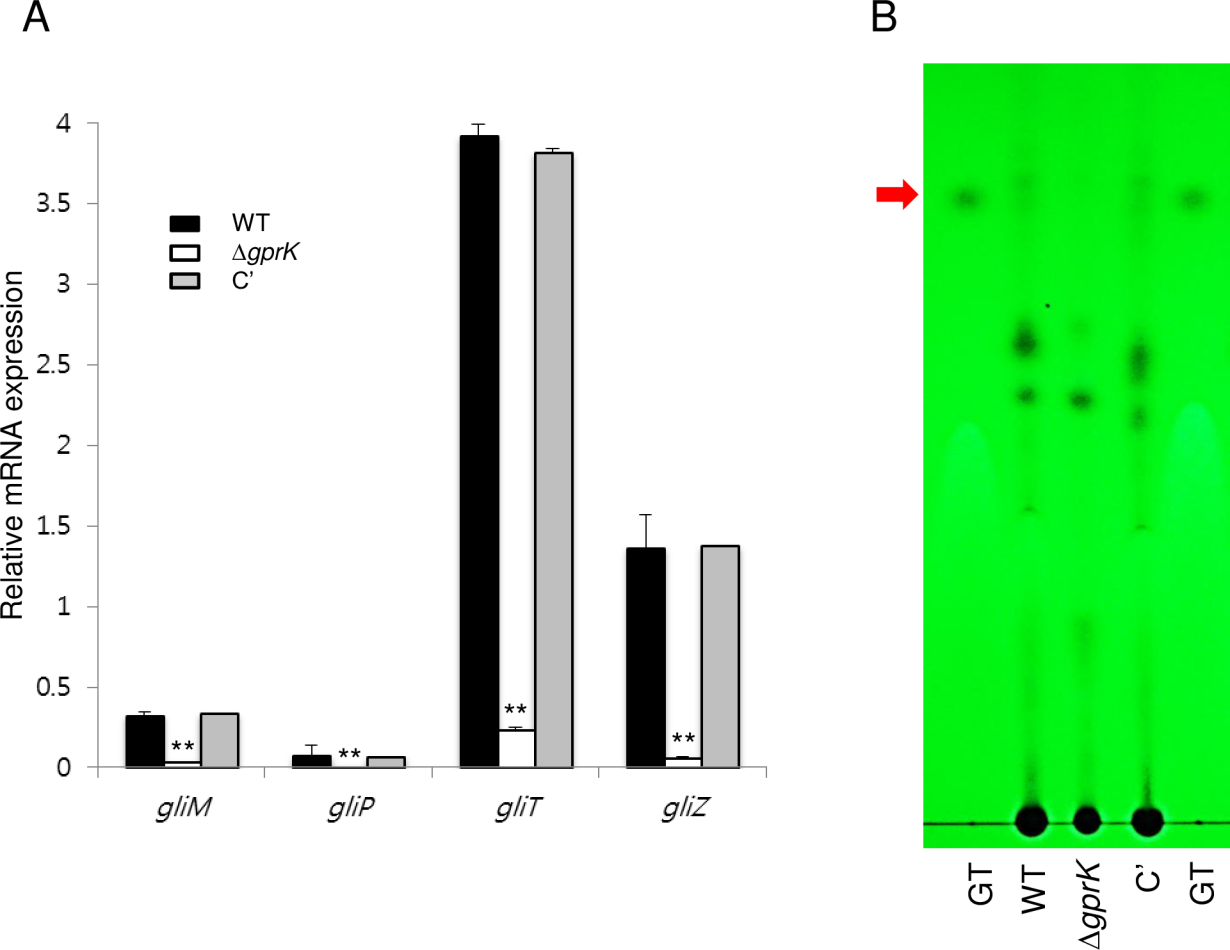
The role of GprK in GT production. (A) qRT-PCR analysis of four GT-related genes in WT, Δ*gprK*, and C' strains. Statistical differences between WT and mutant strains were evaluated with Student's unpaired *t*-test. *p < 0.05 and **p < 0.01. (B) Determination of GT production in WT, Δ*gprK*, and C' strains. The culture supernatant of each strain was extracted with chloroform and subjected to TLC. The arrow indicates the migration position for GT.

We have shown that biosynthesis of the mycotoxin gliotoxin (GT) is in part regulated by the asexual developmental activator BrlA [39]. As the deletion of *gprK* resulted in impaired conidiation and lowered *brlA* expression, we examined whether the Δ*gprK* mutant would be defective in GT production. First, we performed quantitative real time PCR using total RNA of WT, mutant, and C’ strains and analyzed mRNA levels of key GT biosynthetic genes. The mRNA levels of the *gliM*, *gliP*, *gliT*, and *gliZ* genes were significantly lower in the Δ*gprK* strain than in WT and C' strains (Fig 4A). We then assessed levels of GT itself in the three strains, and found that the Δ*gprK* strain produced undetectable amounts of GT (Fig 4B).

**Fig 5:**
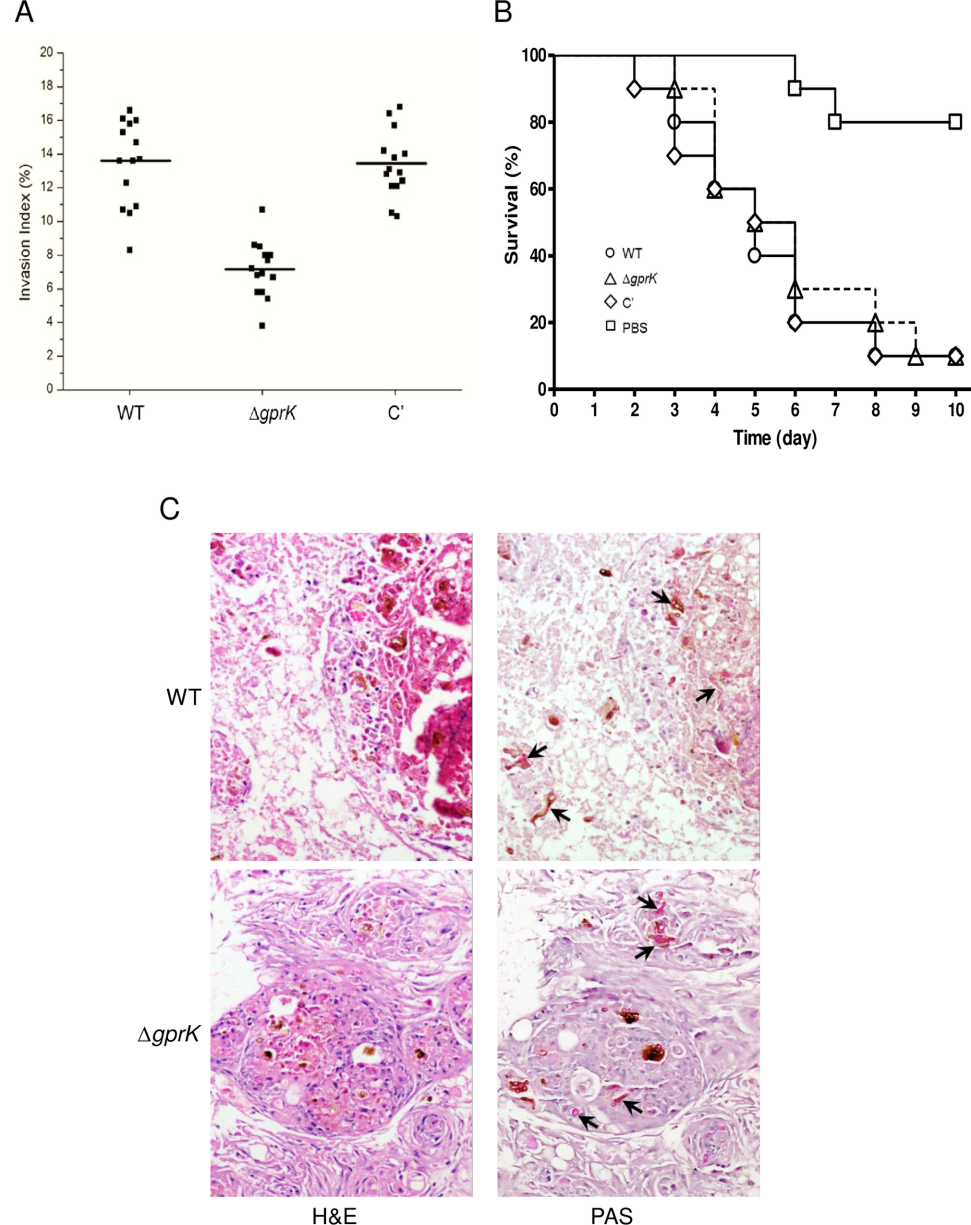
The role of GprK in virulence. (A) Conidia invasion index in type II human alveolar A549 cells inoculated with the Δ*gprK* strain was significantly lower than that of WT. (B) There were no significant differences between WT, Δ*gprK*, and complemented strains as measured by Kaplan-Meier curve survival analysis and log-rank test. (C) Histological analysis of larval tissues infected by WT and Δ*gprK* strains was performed using H&E and PAS staining 24 h post inoculation. Arrows indicated fungal hyphae in infected larval tissues.

We next examined the effect of GprK on virulence using conidia invasiveness in a human cell line and survivability in the *Galleria mellonella* (wax moth) insect model. The ability of conidia to invade host cells is critical for invasive aspergillosis (IA); thus, we tested the conidia invasiveness in the type II human alveolar A549 cell line. The invasion rate of WT conidia was about 13.4% while that of conidia from the Δ*gprK* strain was 7.1%, a 47% reduction in invasiveness in comparison to WT (Fig 5A). To assess survivability, WT and Δ*gprK* strains were inoculated in wax moth larvae, and the larvae survival rate was recorded as a function of time. As shown in Fig 5B, larvae inoculated with either WT or the mutant began to die at day 3 post-inoculation, with the number of survivors continuing to decrease over the course of the experiment. In the first 5 days after infection, about 50% of insects in both strains died. There were no statistically significant differences between WT, Δ*gprK*, and complement strains as measured by *G*. *mellonella* survival (*p* value was 0.7074).

To further understand the fate of *A*. *fumigatus* inoculated into *G*. *mellonella*, infected larvae were fixed in formalin and processed for histopathology. The internal organs were disorganized in the infected larvae, and no nodules or granuloma-like structures were detected in the uninfected control larvae (S3 Fig). Fig 5C showed H&E and PAS-stained sections of infected larvae from WT and Δ*gprK* strains. Both strains induced similar histopathological changes in larvae, and hyphae were observed in sections from larvae infected with either strain. There was evidence of tissue damage in the infected larvae, with pigmented nodules and granulomas associated with hyphae. While most Δ*gprK* fungal hyphae were observed only within nodules, the WT hyphae were distributed extensively (Fig 5C).

### Transcriptome analysis

**Fig 6:**
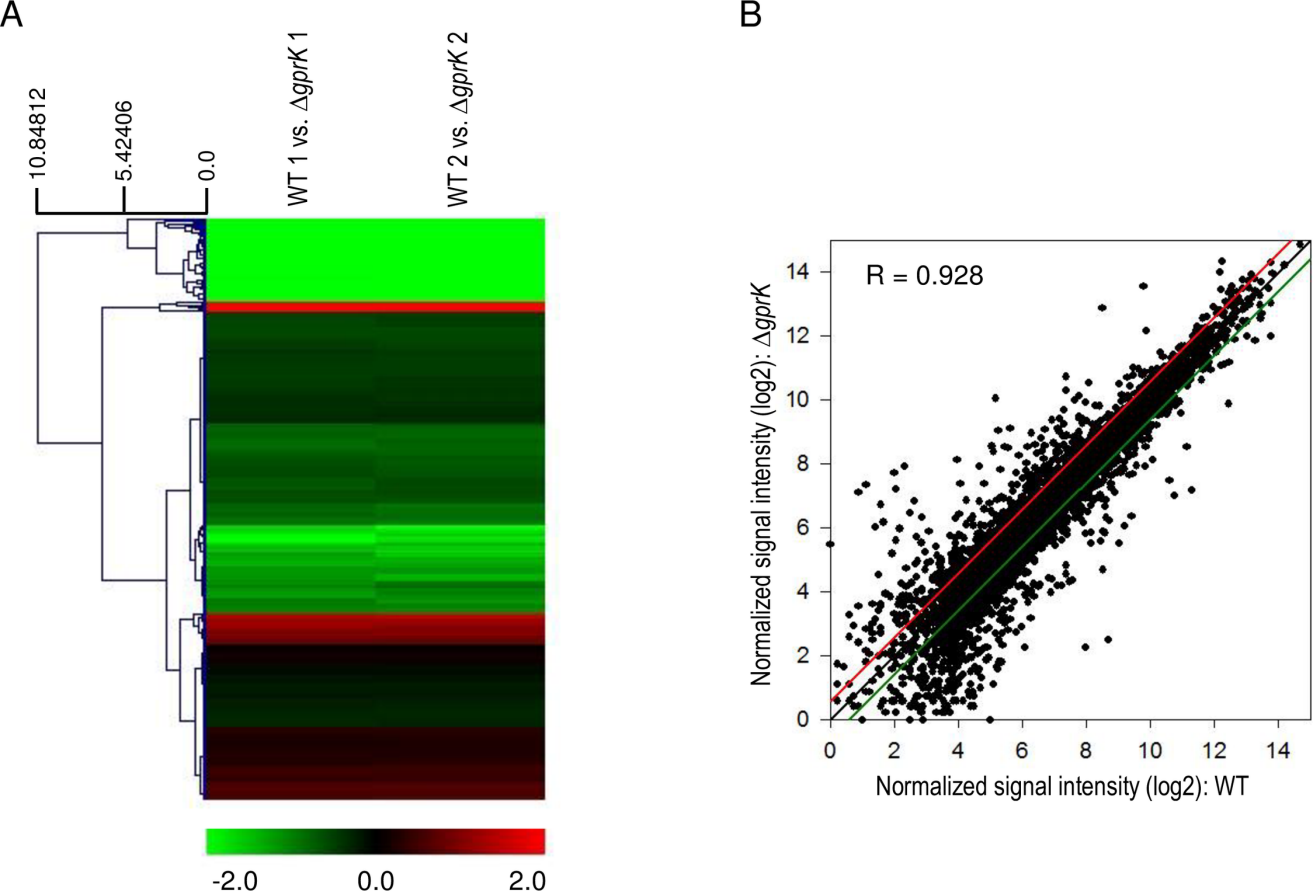
Genome-wide expression correlation between WT and Δ*gprK* strains. (A) Heat map illustration of expression level changes between WT and Δ*gprK* strains. (B) Linear fitted model showing the correlation between overall gene expression for WT and Δ*gprK* strains. The correlation coefficient R is indicated.

To capture the genome-wide expression changes resulting from the absence of GprK, we carried out microarray analysis using Δ*gprK* and WT cells collected at 12 h post asexual-developmental induction. As shown in Fig 6A, the hierarchical clustering heat map based on transcriptome analysis showed that a majority of genes are down-regulated in Δ*gprK* strain compared to WT. Two biological replicates showed a high level of correlation (R = 0.928, Fig 6B). Of the 8,608 probes, 99 genes showed a significant differential expression (at least 1.5 fold, *p*-value < 0.05), of which 17 were up-regulated (higher transcript levels in Δ*gprK* strain than in WT strain) and 82 were down-regulated (S2 Table). Table 1 lists the genes with the highest increase in expression following loss of *gprK*. Using the *Aspergillus* Genome Database (www.aspgd.org) and previous works, we extracted all the available information on the function, localization, genetic pathway, and/or cellular process in which those genes were described or predicted to participate. The majority of the up-regulated genes were predicted to encode for conserved hypothetical proteins, with hydrophobin *rodB* identified as the up-regulated known gene with the maximum fold change in mutant relative to wild-type cells. Most of the down-regulated genes were related to transport (Table 2), including small oligopeptide transporter, nitrate transporter NrtB, ammonium transporter Mep2, MFS transporter, and H^+^/nucleoside cotransporter.

**Table 1:** Up-regulated genes in Δ*gprK* relative to WT.

ID	Locus_tag	Product	Log_2_FC	*p*-value
5750049	AFUA_8G00160	conserved hypothetical protein	5.734	0.0148561
5733376	AFUA_1G17250	conidial hydrophobin RodB	4.317	0.0199937
5736241	AFUA_2G14320	HHE domain protein	4.130	0.0239697
5737657	AFUA_3G02685	conserved hypothetical protein	3.460	0.0087669
5750177	AFUA_8G00740	cytochrome P450, putative	1.757	0.0386637
5744036	AFUA_5G06680	4-aminobutyrate transaminase GatA	1.515	0.0303405
5749834	AFUA_7G06660	conserved hypothetical protein	1.190	0.0038054
5732913	AFUA_1G15330	conserved hypothetical protein	1.149	0.0484174
5739591	AFUA_3G12790	conserved glutamic acid-rich protein	1.096	0.0118515
5740340	AFUA_4G00850	hypothetical protein	1.060	0.0350951
5748050	AFUA_6G12090	HET domain protein	0.979	0.020793
5734405	AFUA_2G04262	C6 transcription factor, putative	0.863	0.0044365
5745470	AFUA_5G13200	thioesterase family protein	0.857	0.0050195
5748459	AFUA_6G14050	FAD binding domain protein	0.835	0.008598
5734686	AFUA_2G05490	HEAT repeat protein	0.714	0.0364938
5742321	AFUA_4G12270	LIM domain protein	0.686	0.0052721
5749217	AFUA_7G03960	thioesterase family protein	0.615	0.0336797

**Table 2:** Top 20 down-regulated genes in Δ*gprK* relative to WT.

ID	Locus_tag	Product	Log_2_FC	*p*-value
5736469	AFUA_2G15240	small oligopeptide transporter, OPT family	-6.058	0.0054359
5741273	AFUA_4G07190	ornithine carbamoyltransferase	-5.442	0.0131424
5746300	AFUA_6G03140	oligopeptide transporter, putative	-4.836	0.0079149
5733915	AFUA_2G02000	conserved hypothetical protein	-4.321	6.404E-05
5743196	AFUA_5G01690	DUF1445 domain protein	-4.293	0.0386637
5733419	AFUA_1G17470	high affinity nitrate transporter NrtB	-4.035	0.0242195
5733093	AFUA_1G16070	conserved hypothetical protein	-3.943	0.0178849
5730314	AFUA_1G01960	conserved hypothetical protein	-3.737	0.0060923
5732457	AFUA_1G13210	uridine permease Fui1, putative	-3.717	0.0137503
5731954	AFUA_1G10930	ammonium transporter (Mep2), putative	-3.426	0.0162459
5743018	AFUA_5G00870	conserved hypothetical protein	-3.307	0.0179706
5730436	AFUA_1G02530	MFS sugar transporter, putative	-3.107	0.0101138
5736911	AFUA_2G17305	hypothetical protein	-3.046	0.0363186
5742705	AFUA_4G14230	MFS transporter, putative	-2.932	0.0228129
5738847	AFUA_3G09390	AMMECR1 family protein	-2.899	0.0389796
5748265	AFUA_6G13190	H+/nucleoside cotransporter	-2.878	0.0455004
5741172	AFUA_4G06620	Glu/Leu/Phe/Val dehydrogenase, putative	-2.634	0.0089898
5745210	AFUA_5G12035	conserved hypothetical protein	-2.617	0.0374917
5743818	AFUA_5G05610	cell cycle control protein Cwf14/Bud31	-2.599	0.0147689
5735341	AFUA_2G09860	purine-cytosine permease	-2.590	0.0298579

## Discussion

*A*. *fumigatus* is an important opportunistic human pathogen that can cause high rates of mortality in immunocompromised humans [6–8]. Most other *Aspergillus* spp. do not cause human disease, so an understanding of how *A*. *fumigatus* differs from others *Aspergillus* spp., both in its virulence and in its ability to respond to different environments, may provide important clues that can lead to better control of and treatments for this pathogen.

Fungi have developed multiple systems to sense extracellular and intracellular signals in order to adapt to their environment. The GPCR gene family represents one of the most important and diverse sensor systems and has been found to play important roles in nutrient sensing and stress responses in many fungal species [5, 9, 12, 36, 40–44].

The 15 predicted *A*. *fumigatus* GPCRs fall into 9 classes as previously described [6]. GprK belongs to class VI of fungal GPCRs, which are characterized by the presence of both a 7-TM and a cytoplasmic RGS domain. A GPCR-RGS hybrid was first discovered in *Arabidopsis thaliana* (AtRGS1), which modulates plant cell proliferation via the Gpa1 Gα subunit [45]. Unlike canonical GPCRs, AtRGS1 does not trigger the GDP-GTP exchange by a G protein. Instead, AtRGS1 interacts with the constitutively active Gα subunit, resulting in hydrolysis of GTP and subsequent deactivation of the G protein [45–47]. This type of GPCR has since been found in several species of filamentous fungi [6, 48–50]. Similar to other filamentous ascomycetes, *A*. *fumigatus* possess only one GprK (AFUA_4G01350). However, the exact functions for GprK have been elucidated in only two fungal species. GprK of *A*. *flavus* is involved in germination, nutrient sensing, toxin production, and pH stress response [38]. The *gprK* deficient mutant showed 50% of the WT germination rate and was impaired in growth on galactose and xylose. The Δ*gprK* mutant was more sensitive than the wild type strain to Congo red, hyperosmotic conditions, and acidic and alkaline pHs [38]. The most-studied filamentous fungus, *N*. *crassa*, also possesses a GprK-like protein, Gpr-7. The Δ*gpr-7* mutant was resistant to FK506, which inhibits the phosphatase calcineurin. However Gpr-7 did not appear to be essential for hyphal growth, asexual development, or sexual differentiation [51].

In the presence of external signals, GPCRs are sensitized and interact with heterotrimeric G proteins, resulting in the dissociation of GTP-Gα from the Gβγ heterodimer. Activated G protein mediated signaling is transmitted *via* various downstream components, including cAMP-dependent protein kinase (PKA) [1, 2, 4, 52]. In contrast to GprK of *A*. *flavus* and Gpr-7 of *N*. *crassa*, deletion of *gprK* caused enhanced activation of PKA, resulting in restricted asexual sporulation, reduced expression of key asexual regulators, and hyper-active conidial germination (Figs 1 & 2). These observations led to the hypothesis that a role of GprK is to negatively regulate a cAMP-dependent PKA pathway.

While a nutrient-sensing GPCR has not been identified in the *A*. *fumigatus*, GprK may be involved in carbon source sensing. Indeed, a majority of down-regulated genes in the Δ*gprK* mutant were transport-related genes (Table 2). GprK also appears to be involved in response to oxidative stress. The *gprK* deletion mutant was hypersensitive to hydrogen peroxide and menadione (Fig 3) but not to cell wall stressors, hyperosmotic conditions, and pH stresses (S2 Fig). Activated G-protein mediated signaling is transmitted through mitogen-activated protein kinase (MAPK) pathways [1, 2, 4]. Stress MAPK (SakA) is involved in stress signal transduction and is required for spore stress resistance and survival [53, 54]. It interacts with ATF/CREB transcription factor AtfA, and the SakA-AtfA interaction regulates gene expression during oxidative stress in *A*. *nidulans* [54]. It has been demonstrated that accumulation of spore-specific *catA* mRNA was dependent on SakA and AtfA [54], and CatA activity in Δ*sakA* conidia was lower than in WT conidia [53]. The *sod1* and *sod2* of *A*. *fumigatus* were highly expressed in conidia, and Δ*sod1* and Δ*sod2* mutants showed hyper-sensitivity to menadione, which produces intracellular superoxide radicals [55]. The expression of *sakA* and *atfA* mRNA was significantly reduced in the Δ*gprK* mutant conidia and CatA, SOD1, and SOD2 activities were also drastically reduced in Δ*gprK* strain (Fig 3). Based on our results, we propose that GprK positively regulates the SakA and AtfA, and the SakA-AtfA controlled expression of conidia-specific catalase and SODs.

The GT biosynthetic gene cluster is composed of numerous genes. The *gliM* gene is predicted to encode an *o*-methyltransferase [56], and GliP catalyzes the first steps of GT biosynthesis [57]. The *gliT* gene encodes an oxidoreductase of the GT biosynthetic cluster [58, 59] and GliZ, a Zn2Cys6 transcription factor, is responsible for general GT induction and regulation [60, 61]. Expression of GT biosynthetic genes and GT production in Δ*gprK* strain was severely reduced compared to WT and C' strain (Fig 4), suggesting that GprK regulates GT synthesis positively, likely by activating the asexual developmental regulator *brlA*. GT is an important virulence determinant of *A*. *fumigatus* and the Δ*gliP* strain was significantly less virulent and deletion of *gliP* abrogated GT production [62].

The ability of conidia to invade host cells is also a critical virulence determinant. Toxins that are secreted by conidia, such as GT, can affect ciliary movement of the host’s bronchial epithelial cells and increase the chance of colonization [63]. The invasion rate of conidia for the Δ*gprK* strain was significantly reduced (about 47% of WT), suggesting that GprK plays a role in virulence. A previous study also found a positive correlation between the level of GT production and pathogenicity level in greater wax moths [64]. Larvae infected with the low GT-producing strains exhibited reduced mortality, while the high mortality of larvae infected with the high GT producing strain suggested that GT production was a significant contributor to the pathogenicity of *A*. *fumigatus* in *G*. *mellonella*. Survival analysis of *G*. *mellonella* larvae challenged with the Δ*gprK* mutant and WT have revealed that the mortality level of Δ*gprK* strain was similar to that of WT strain (Fig 5B). Microscopically, we observed the presence of granuloma-like structures in infected larval tissue, while no nodules or granuloma-like structures were detected in the uninfected control larvae (S3 Fig). While most of fungal hyphae of the Δ*gprK* mutant were observed only within nodules of infected larvae, the hyphae of the WT were distributed extensively (Fig 5C).

It was revealed that the hydrophobin *rodB* was up-regulated in the Δ*gprK* mutant with the maximum fold change in our microarray analysis (Table 1). RodB, which is specific to *A*. *fumigatus*, is involved in building the conidial outer cell wall, but does not protect conidia against killing by lung alveolar macrophages [65]. Most of the genes down-regulated in the Δ*gprK* mutant were related to transport, such as the small oligopeptide transporter, nitrate transporter NrtB, ammonium transporter Mep2, MFS transporter, and H^+^/nucleoside cotransporter (Table 2). *A*. *nidulans* possesses two nitrate transporters, NrtA and NrtB [66, 67], and nitrate transport has been shown to be proton-dependent [68]. The NrtA and NrtB nitrate transporters are paralogous members of the major facilitator superfamily (MFS) and the NrtB transporter may be more effective at scavenging nitrate from low external concentrations [66]. Ammonium transporter Mep2 is a transporter for ammonium to use as a nitrogen source and under ammonium limitation, Mep2 acts as an ammonium sensor, generating a signal that leads to pseudohyphal growth in *S*. *cerevisiae* [69]. These results suggest that the Δ*gprK* mutant fails to sense external nutrients effectively, which may be associated with down-regulation of a majority of transporters.

## Supporting Information

S1 Fig Fifteen predicted GPCRs in *A*. *fumigatus* and a phylogenetic tree of GprK-like proteins in filamentous fungi. (A) Predicted *A*. *fumigatus* GPCRs are presented schematically using SMART (http://smart.embl-heidelberg.de) with the number of TMs shown in parenthesis. Small pink rectangles are indicated low complexity regions. (B) The phylogenetic tree is constructed based on the matrix of pair-wise distances between sequences. NFIA 043940: hypothetical protein of *N*. *fischeri* NRRL 181, ACLA 067510: conserved hypothetical protein of *A*. *clavatus* NRRL 1, ANID 07795.1: conserved hypothetical protein of *A*. *nidulans* FGSC A4, ATEG 08180.1: conserved hypothetical protein of *A*. *terreus* NIH2624, AO090103000244: hypothetical protein of *A*. *oryzae* RIB40, AFL2G 12145.2: conserved hypothetical protein of *A*. *flavus* NRRL3357, ASPNIDRAFT 131536: hypothetical protein of *A*. *niger* ATCC 1015. (C) Alignment of the GprK homologs of *A*. *fumigatus* (Afu4g01350), *A*. *niger* (An04g07760), and *A*. *nidulans* (AN7795). The identical amino acids are marked by shades. ClustalW (http://align.genome.jp/) was used for the alignment.
(PPTX)

S2 Fig Sensitivity of mutant lacking *gprK* towards cell wall stress, osmotic stress, and different pH levels. About 1×10^6^ spores of each strain was spotted on to YG agar plates supplemented with indicated amount of Congo red (CR), calcofluor white (CFW), caspofungin (CAS), sodium chloride, or sorbitol and incubated at 37°C for 48 h. Additional plates contained media buffered to three different pHs before autoclaving. Statistical significance was determined by a Student’s *t*-test, with *p < 0.05 and **p < 0.01.
(PPTX)

S3 Fig Histological analysis of larval tissues infected by *A*. *fumigatus*. (A) Uninfected control larvae and (B) infected larvae with WT. Note that the internal organs were not clear in the infected larvae and no nodules or granuloma-like structures were detected in the uninfected control larvae. Arrows indicate nodules or granulomas-like structures. c: cuticle, a: adipose tissue, g: gastrointestinal tract.
(PPTX)

S1 Table Oligonucleotides used in this study. (DOC)

S2 Table Significantly differentially expressed genes in Δ*gprK* relative to WT (≥1.5 fold, *p*-value < 0.05). (DOC)
