# Supplementary material for: Characterization of gprK Encoding a Putative Hybrid G-Protein-Coupled Receptor in Aspergillus fumigatus
Source: PLoS One. 2016 Sep 1;11(9):e0161312. doi: 10.1371/journal.pone.0161312 (PMC5008803; doi:10.1371/journal.pone.0161312)
Supplement: S1 Fig — (A) Predicted A. fumigatus GPCRs are presented schematically using SMART (http://smart.embl-heidelberg.de) with the number of TMs shown in parenthesis. Small pink rectangles are indicated low complexity regions. (B) The phylogenetic tree is constructed based on the matrix of pair-wise distances between sequences. NFIA 043940: hypothetical protein of N. fischeri NRRL 181, ACLA 067510: conserved hypothetical protein of A. clavatus NRRL 1, ANID 07795.1: conserved hypothetical protein of A. nidulans FGSC A4, ATEG 08180.1: conserved hypothetical protein of A. terreus NIH2624, AO090103000244: hypothetical protein of A. oryzae RIB40, AFL2G 12145.2: conserved hypothetical protein of A. flavus NRRL3357, ASPNIDRAFT 131536: hypothetical protein of A. niger ATCC 1015. (C) Alignment of the GprK homologs of A. fumigatus (Afu4g01350), A. niger (An04g07760), and A. nidulans (AN7795). The identical amino acids are marked by shades. ClustalW (http://align.genome.jp/) was used for the alignment. (PPTX) [file pone.0161312.s001.pptx]

## Slide 1
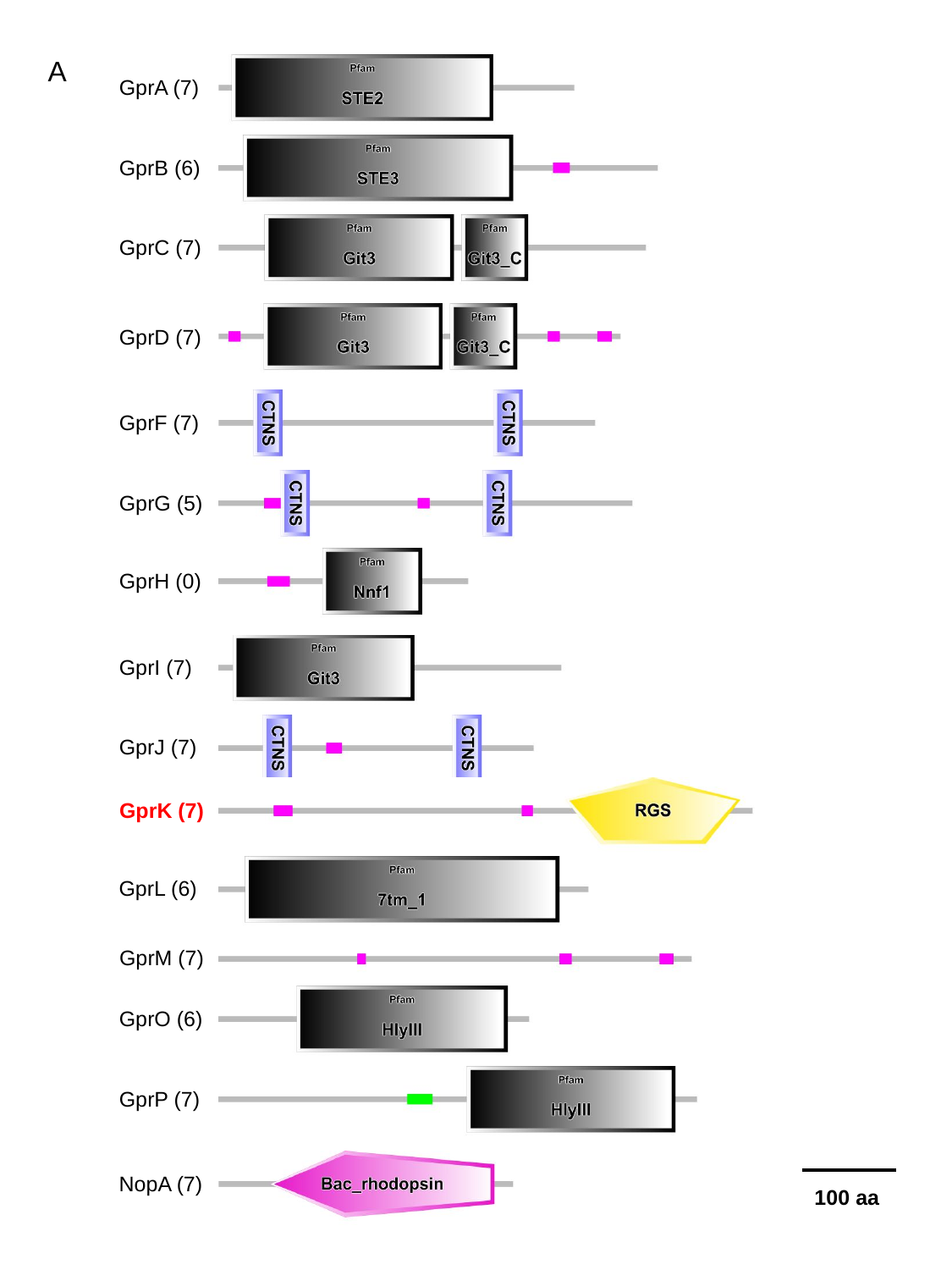

A
100 aa
GprA (7)
GprB (6)
GprC (7)
GprD (7)
GprF (7)
GprG (5)
GprH (0)
GprI (7)
GprJ (7)
GprK (7)
GprL (6)
GprM (7)
GprO (6)
GprP (7)
NopA (7)

## Slide 2
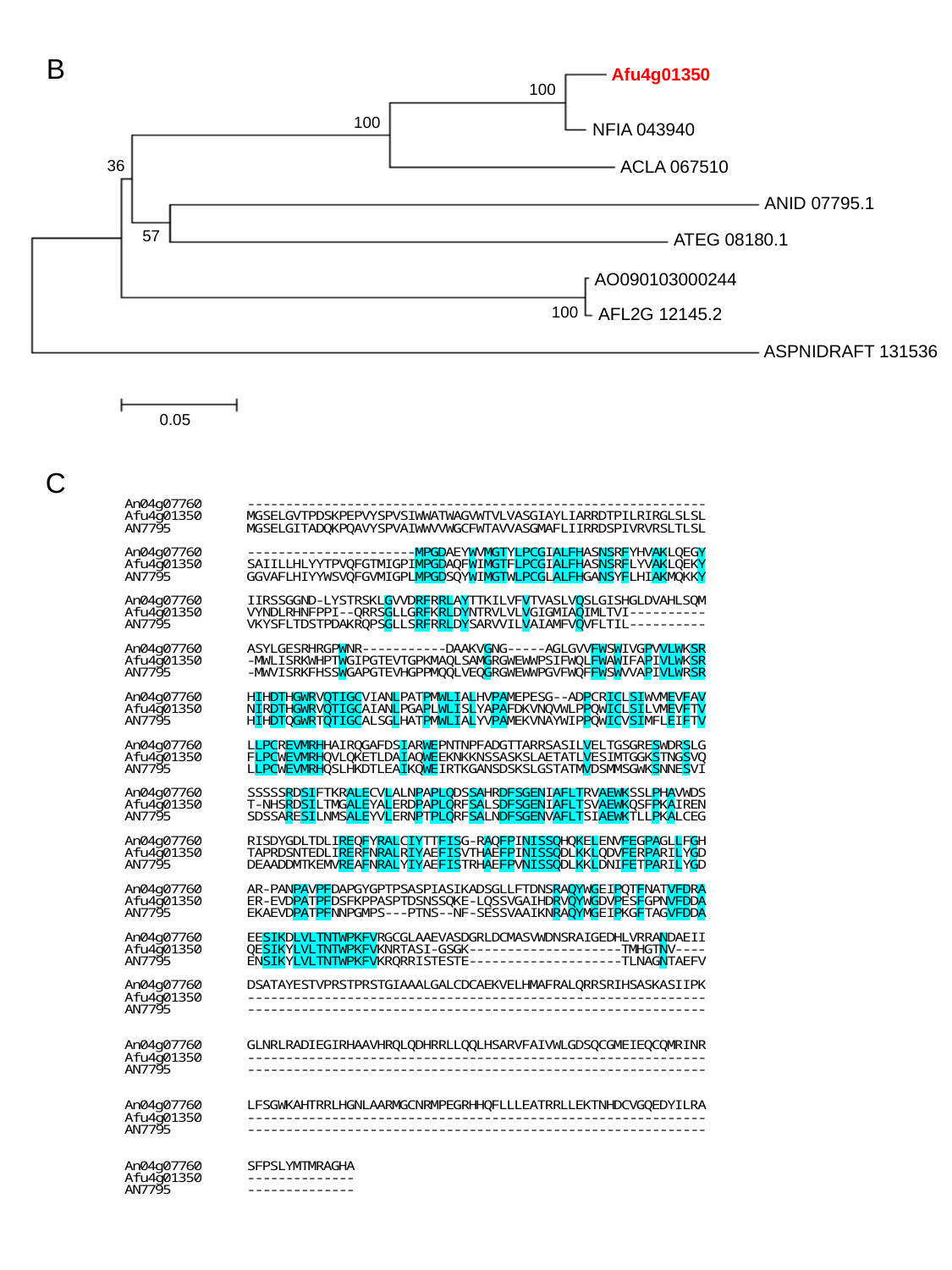

Afu4g01350
NFIA 043940
ACLA 067510
ANID 07795.1
ATEG 08180.1
AO090103000244
AFL2G 12145.2
ASPNIDRAFT 131536
0.02
100
100
36
57
100
0.05
B
C
