# Supplementary material for: Characterization of gprK Encoding a Putative Hybrid G-Protein-Coupled Receptor in Aspergillus fumigatus
Source: PLoS One. 2016 Sep 1;11(9):e0161312. doi: 10.1371/journal.pone.0161312 (PMC5008803; doi:10.1371/journal.pone.0161312)
Supplement: S2 Fig — About 1×10^6^ spores of each strain was spotted on to YG agar plates supplemented with indicated amount of Congo red (CR), calcofluor white (CFW), caspofungin (CAS), sodium chloride, or sorbitol and incubated at 37°C for 48 h. Additional plates contained media buffered to three different pHs before autoclaving. Statistical significance was determined by a Student’s t-test, with *p < 0.05 and **p < 0.01. (PPTX) [file pone.0161312.s002.pptx]

## Slide 1
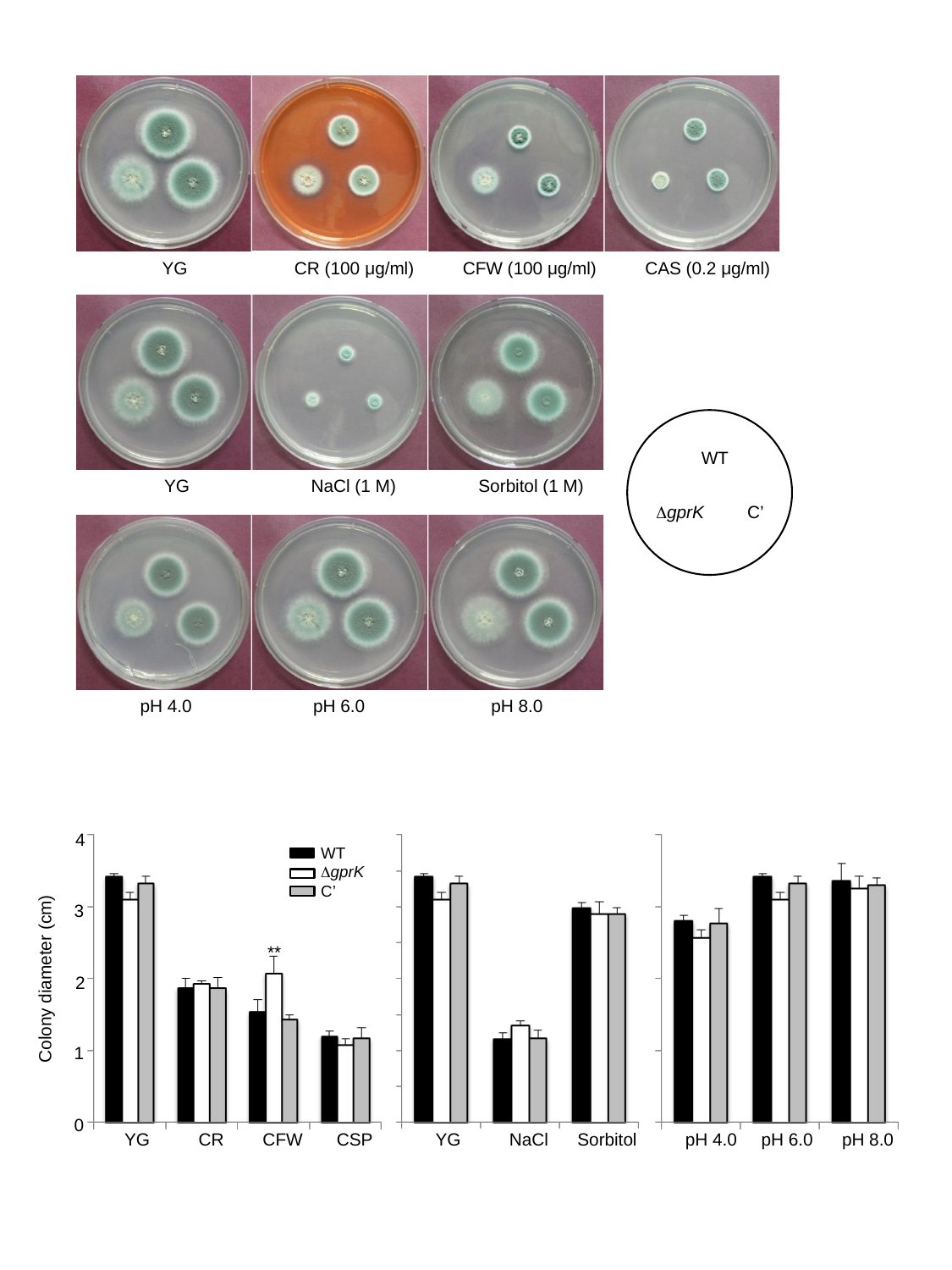

YG CR (100 μg/ml) CFW (100 μg/ml) CAS (0.2 μg/ml)
 YG NaCl (1 M) Sorbitol (1 M)
WT
DgprK
C’
pH 4.0 pH 6.0 pH 8.0
4
WT
DgprK
C’
Colony diameter (cm)
 YG CR CFW CSP YG NaCl Sorbitol pH 4.0 pH 6.0 pH 8.0
3
**
2
1
0
