# Supplementary figures and images for: Characterization of gprK Encoding a Putative Hybrid G-Protein-Coupled Receptor in Aspergillus fumigatus
Source: PLoS One. 2016 Sep 1;11(9):e0161312. doi: 10.1371/journal.pone.0161312 (PMC5008803; doi:10.1371/journal.pone.0161312)

## Slide 1
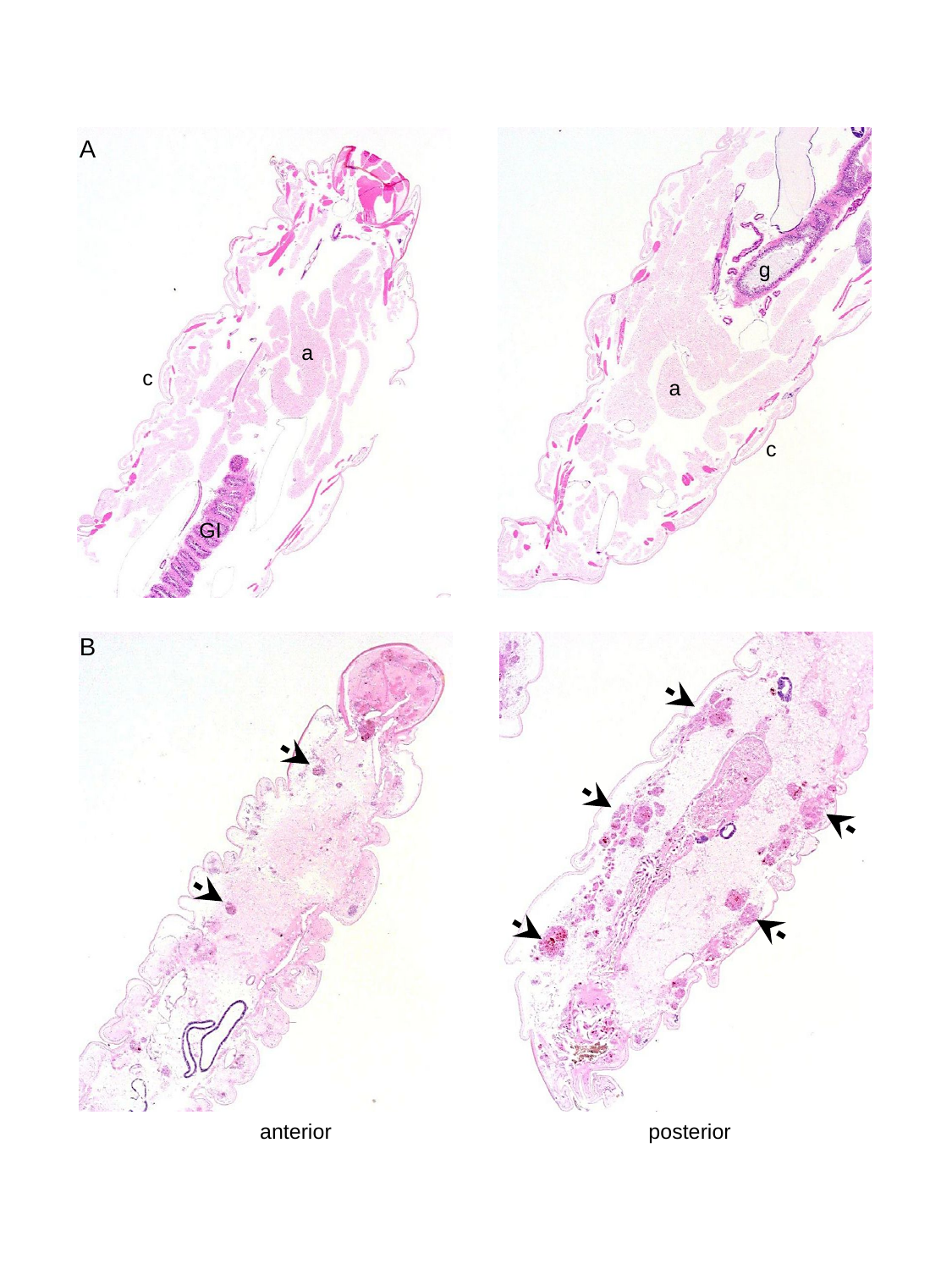

A
g
a
c
a
c
GI
B
posterior
anterior

Supplement: S3 Fig — (A) Uninfected control larvae and (B) infected larvae with WT. Note that the internal organs were not clear in the infected larvae and no nodules or granuloma-like structures were detected in the uninfected control larvae. Arrows indicate nodules or granulomas-like structures. c: cuticle, a: adipose tissue, g: gastrointestinal tract. (PPTX) [file pone.0161312.s003.pptx]
