# Supplementary material for: Characterization of gprK Encoding a Putative Hybrid G-Protein-Coupled Receptor in Aspergillus fumigatus
Source: PLoS One. 2016 Sep 1;11(9):e0161312. doi: 10.1371/journal.pone.0161312 (PMC5008803; doi:10.1371/journal.pone.0161312)
Supplement: S1 Table — (DOC) [file pone.0161312.s004.doc]

S1 Table. Oligonucleotides used in this study.

| Name | Sequence (5'→3')^a^ | Purpose |
| --- | --- | --- |
| oligo346  oligo347  oligo303  oligo304  oligo305  oligo306  oligo307  oligo308  oligo256  oligo257  oligo434  oligo435  oligo508  oligo509  oligo514  oligo515  oligo275  oligo276  oligo277  oligo278  oligo260  oligo261  oligo262  oligo263  oligo689  oligo690  oligo691  oligo692  oligo693  oligo694  oligo271  oligo272  oligo109  oligo110  oligo295  oligo296  oligo297  oligo298  oligo299  oligo300  oligo785  oligo786  oligo787  oligo788  oligo730  oligo731  oligo295  oligo886  oligo888  oligo887  oligo889  oligo890 | CCATGTGTGTCGAGTCCTTC  GAACGTACAGCAACAGTCTGG  GCTACCACTCTGCATCCTCA  TACGAGCTCCAGCATGATTC  ACGGCAGGAAGTTGTCTTCT'  CTGTCAGCGACTTGTTGGAT  ATGAACCAGCCAGGTACTCC  GTTGGTCCCTTGTGGAAAGT  TTCCAAGCAGAGCTTGTCAC  CCAGGTTCTTTGCACTTGAA  CCACCACCTACAACAACAGC  TGTGAAGACGCATGATGAGA  GAGAATACCCTGCGGTTTGT  GGATCCTCTTCTTGGGATCA  ACTCCGAGGATGAGGATGAC  GACCCTGTTCCTTTCCAAGA  AGCCACGGCTCTTCTACAAT  GTTCTTCACCACGGGAGATT  ATTCCAGAGGAGAAGCAGGA  GAGCTCTCCAATCACACGAA  TCATTGCTGTCCTCCGTGGTG  GGTCGTTGCCCTTGATGTTCC  GGTCAGTTCCAGCCTCTTCTTG  CTTCTCCACAGCCTTCCAGTTG  AAACCCCTGTGAATGCAGAC  CCCCTTGAGATGAAAGGTGA  CGATCTGTACCCCAACGAGT  TTCTGGAACTTTGCCAGCTT  ACTCCACCATCCAGTTCCAG  TCCGAGTATCCCTCGATGTC  AAATCCATCACATCCACCCT  GGTTGTTCATGGTCAGTTGC  GCAATGTAAAGCTAACGTGCGTG  TGCCTTTAAGCTTCGGGTAGAG  CAGCCTTCCTCGAGAATCAG  *TTTGTAGGCTTTGGGCTGTTC*ACAAGTGTGGTTGAAGCAACAGGA  GTCGTCGTCTTCAGCTCCTC  *CTGATCTACCCCTTGGAACGCAGCA*ACTCCGGACTCATGTTGGAT  GTGATCAGGGCTACCAAGGA  AAGGGTAACGCCTTTGGTGT  GCACGATGTAGAACCCGGCA  *AAACAAAGATGCAAGAGCGGC*CCA ACATGAGTCCGGAGTCAAGG  CCTTGACTCCGGACTCATGTTGG*GCCGCTCTTGCATCTTTGTTT*  GGGTTAGCAATGGGGTCCCA  GCCGCTCTTGCATCTTTGTTT  AATTGATTACGGGATCCCATTGG  CAGCCTTCCTCGAGAATCAG  *AAACAAAGATGCAAGAGCGGC*TCCTACGCGTCCATTAGCGA  GAAGGGAGCGAGCGCTTCAA  TCGCTAATGGACGCGTAGGA*GCCGCTCTTGCATCTTTGTTT*  AATTGAATTCTAGCAATGGGGTCCCACCA  AATTAAGCTTGAAGGGAGCGAGCGCTTCAA | 5' *ef1* for qRT-PCR normalization  3' *ef1* for qRT-PCR normalization  5' *abaA* for qRT-PCR  3' *abaA* for qRT-PCR  5' *wetA* for qRT-PCR  3' *wetA* for qRT-PCR  5' *vosA* for qRT-PCR  3' *vosA* for qRT-PCR  5' *brlA* for qRT-PCR  3' *brlA* for qRT-PCR  5' *pkaC1* for qRT-PCR  3' *pkaC1* for qRT-PCR  5' *sakA* for qRT-PCR  3' *sakA* for qRT-PCR  5' *atfA* for qRT-PCR  3' *atfA* for qRT-PCR  5' *cat1* for qRT-PCR  3' *cat1* for qRT-PCR  5' *catA* for qRT-PCR  3' *catA* for qRT-PCR  5' *sod1* for qRT-PCR  3' *sod1* for qRT-PCR  5' *sod2* for qRT-PCR  3' *sod2* for qRT-PCR  5' *gliP* for qRT-PCR  3' *gliP* for qRT-PCR  5' *gliM* for qRT-PCR  3' *gliM* for qRT-PCR  5' *gliT* for qRT-PCR  3' *gliT* for qRT-PCR  5' *gliZ* for qRT-PCR  3' *gliZ* for qRT-PCR  5' *AnipyrG* marker  3' *AnipyrG* marker  5' flanking region of *gprK*  5' *gprK* with *AnipyrG* tail  3' flanking region of *gprK*  3' *gprK* with *AnipyrG* tail  5' nested of *gprK*  3' nested of *gprK*  5' *gprK* for complementation  3' *gprK* with *ptrA* for complementation  5' *ptrA* with *gprK* for complementation  5' nest *gprK*  5' of *ptrA*  3' nest of *ptrA*  5' flanking region of *gprK* (GPCR)  5' *gprK* (GPCR) with *ptrA* tail  3' flanking region of *gprK* (GPCR)  3' *gprK* (GPCR) with *ptrA* tail  5' nested of *gprK* (GPCR)  3' nested of *gprK* (GPCR) |

^a^ Tail sequence is in italic.
