# Supplementary material for: Characterization of gprK Encoding a Putative Hybrid G-Protein-Coupled Receptor in Aspergillus fumigatus
Source: PLoS One. 2016 Sep 1;11(9):e0161312. doi: 10.1371/journal.pone.0161312 (PMC5008803; doi:10.1371/journal.pone.0161312)
Supplement: S2 Table — (DOC) [file pone.0161312.s005.doc]

| S2 Table. Significant differentially expressed genes in Δ*gprK* relative to WT (1.5 fold, *p*-value < 0.05). | | | | |
| --- | --- | --- | --- | --- |
| ID | Fold change | *p*-value | Product | Locus_tag |
| 5750049 | 53.237 | 0.014856053 | conserved hypothetical protein | AFUA_8G00160 |
| 5733376 | 19.942 | 0.01999368 | conidial hydrophobin RodB | AFUA_1G17250 |
| 5736241 | 17.517 | 0.023969652 | HHE domain protein | AFUA_2G14320 |
| 5737657 | 11.011 | 0.008766922 | conserved hypothetical protein | AFUA_3G02685 |
| 5750177 | 3.381 | 0.03866365 | cytochrome P450, putative | AFUA_8G00740 |
| 5744036 | 2.858 | 0.030340478 | 4-aminobutyrate transaminase GatA | AFUA_5G06680 |
| 5749834 | 2.282 | 0.003805372 | conserved hypothetical protein | AFUA_7G06660 |
| 5732913 | 2.219 | 0.048417374 | conserved hypothetical protein | AFUA_1G15330 |
| 5739591 | 2.138 | 0.011851501 | conserved glutamic acid-rich protein | AFUA_3G12790 |
| 5740340 | 2.085 | 0.035095092 | hypothetical protein | AFUA_4G00850 |
| 5748050 | 1.972 | 0.02079304 | HET domain protein | AFUA_6G12090 |
| 5734405 | 1.820 | 0.004436496 | C6 transcription factor, putative | AFUA_2G04262 |
| 5745470 | 1.812 | 0.005019535 | thioesterase family protein | AFUA_5G13200 |
| 5748459 | 1.785 | 0.008598034 | FAD binding domain protein | AFUA_6G14050 |
| 5734686 | 1.641 | 0.036493834 | HEAT repeat protein | AFUA_2G05490 |
| 5742321 | 1.609 | 0.005272116 | LIM domain protein | AFUA_4G12270 |
| 5749217 | 1.532 | 0.03367967 | thioesterase family protein | AFUA_7G03960 |
| 5738558 | 0.663 | 0.024189087 | C2H2 transcription factor (Ace1), putative | AFUA_3G08010 |
| 5742712 | 0.656 | 0.038030602 | O-methyltransferase, putative | AFUA_4G14240 |
| 5732135 | 0.656 | 0.015019651 | conserved hypothetical protein | AFUA_1G11760 |
| 5750706 | 0.654 | 0.01750637 | glucosamine-6-phosphate deaminase, putative | AFUA_8G04070 |
| 5741019 | 0.652 | 0.04105619 | malonyl CoA-acyl carrier protein transacylase | AFUA_4G05850 |
| 5736535 | 0.650 | 0.026912902 | conserved hypothetical protein | AFUA_2G15580 |
| 5734617 | 0.649 | 0.003262548 | ER membrane protein (Pkr1), putative | AFUA_2G05200 |
| 5746508 | 0.641 | 0.04568402 | conserved hypothetical protein | AFUA_6G04060 |
| 5732492 | 0.631 | 0.025867581 | aflatoxin B1-aldehyde reductase GliO-like protein | AFUA_1G13370 |
| 5748203 | 0.630 | 0.01919752 | hypothetical protein | AFUA_6G12880 |
| 5738771 | 0.625 | 0.000722202 | regulatory protein SUAPRGA1 | AFUA_3G09030 |
| 5744553 | 0.611 | 0.021428581 | cytochrome c oxidase assembly protein (Pet191) | AFUA_5G08965 |
| 5736510 | 0.600 | 0.017289713 | sorbitol/xylulose reductase Sou1-like, putative | AFUA_2G15430 |
| 5732797 | 0.587 | 0.000364646 | mitochondrial inheritance component mdm12 | AFUA_1G14790 |
| 5738818 | 0.585 | 0.032550927 | cell wall glucanase, putative | AFUA_3G09250 |
| 5739092 | 0.584 | 0.021770597 | PQ loop repeat protein | AFUA_3G10470 |
| 5743327 | 0.578 | 0.019755462 | potassium ion transporter (Trk1), putative | AFUA_5G02290 |
| 5742245 | 0.577 | 0.002845477 | alkaline serine protease Alp1 | AFUA_4G11800 |
| 5734602 | 0.569 | 0.029073814 | ankyrin repeat protein | AFUA_2G05140 |
| 5744016 | 0.564 | 0.03759511 | SNF2 family helicase, putative | AFUA_5G06590 |
| 5735104 | 0.559 | 0.011810607 | nuclear distribution protein NudE | AFUA_2G08690 |
| 5730776 | 0.554 | 0.008486982 | C6 finger domain protein, putative | AFUA_1G04140 |
| 5731234 | 0.548 | 0.000499205 | SNF7 family protein | AFUA_1G06420 |
| 5745824 | 0.542 | 0.039306376 | conserved serine-proline rich protein | AFUA_5G14950 |
| 5750873 | 0.539 | 0.027463047 | MFS transporter, putative | AFUA_8G04850 |
| 5744450 | 0.527 | 0.046042204 | phosphatidylinositol 3,5-bisphosphate-binding protein | AFUA_5G08530 |
| 5745212 | 0.510 | 0.01854267 | conserved hypothetical protein | AFUA_5G12040 |
| 5750769 | 0.500 | 0.010855513 | GPI anchored protein, putative | AFUA_8G04370 |
| 5739734 | 0.486 | 0.042227417 | stomatin family protein | AFUA_3G13440 |
| 5743491 | 0.477 | 0.009521714 | C6 transcription factor, putative | AFUA_5G03030 |
| 5737368 | 0.474 | 0.021378232 | class II aldolase/adducin domain protein | AFUA_3G01330 |
| 5736645 | 0.473 | 0.032495543 | conserved hypothetical protein | AFUA_2G16060 |
| 5747107 | 0.469 | 0.045307674 | hypothetical protein | AFUA_6G07790 |
| 5742996 | 0.451 | 0.023694528 | integral membrane protein, putative | AFUA_5G00770 |
| 5746724 | 0.449 | 0.03733077 | polysaccharide deacetylase family protein | AFUA_6G05030 |
| 5732164 | 0.446 | 0.005328061 | PQ loop repeat protein | AFUA_1G11900 |
| 5744876 | 0.438 | 0.002082489 | riboflavin aldehyde-forming enzyme | AFUA_5G10470 |
| 5730615 | 0.435 | 0.01963565 | conserved hypothetical protein | AFUA_1G03370 |
| 5749117 | 0.432 | 0.006898555 | mRNA processing protein (Mss51), putative | AFUA_7G02470 |
| 5743752 | 0.420 | 0.018373925 | arginine transporter, putative | AFUA_5G04260 |
| 5731386 | 0.417 | 0.02958652 | SPX domain protein | AFUA_1G07250 |
| 5745339 | 0.405 | 0.039696854 | hypothetical protein | AFUA_5G12630 |
| 5740077 | 0.384 | 0.010424946 | TAM domain methyltransferase, putative | AFUA_3G14920 |
| 5733109 | 0.372 | 0.045335237 | C6 transcription factor, putative | AFUA_1G16160 |
| 5741276 | 0.355 | 0.024434568 | conserved hypothetical protein | AFUA_4G07200 |
| 5730077 | 0.330 | 0.008908909 | hypothetical protein | AFUA_1G00790 |
| 5747635 | 0.325 | 0.025646003 | MFS monocarboxylate transporter, putative | AFUA_6G10100 |
| 5736672 | 0.306 | 0.000966407 | C6 finger domain protein, putative | AFUA_2G16160 |
| 5748901 | 0.303 | 0.007168942 | MFS peptide transporter Ptr2, putative | AFUA_7G01490 |
| 5737648 | 0.303 | 0.0282624 | nucleoside-diphosphate-sugar epimerase family | AFUA_3G02640 |
| 5738216 | 0.277 | 0.01929953 | COX1 assembly protein Shy1, putative | AFUA_3G06340 |
| 5737227 | 0.272 | 0.04047086 | short-chain dehydrogenase/reductase, putative | AFUA_3G00750 |
| 5743118 | 0.269 | 0.023479756 | C6 transcription factor, putative | AFUA_5G01272 |
| 5749290 | 0.267 | 0.024509076 | amino acid permease (Gap1), putative | AFUA_7G04290 |
| 5746664 | 0.259 | 0.013592061 | UPF0220 domain protein | AFUA_6G04760 |
| 5738572 | 0.237 | 0.005755868 | C6 transcription factor (OTam), putative | AFUA_3G08050 |
| 5749537 | 0.222 | 0.030854648 | conserved hypothetical protein | AFUA_7G05300 |
| 5731308 | 0.210 | 0.026767261 | Nulp1-pending protein | AFUA_1G06850 |
| 5742571 | 0.196 | 0.021124762 | thiol methyltransferase, putative | AFUA_4G13570 |
| 5737163 | 0.193 | 0.040871404 | F-box domain protein | AFUA_3G00440 |
| 5741875 | 0.190 | 0.030245118 | ABC multidrug transporter Mdr2 | AFUA_4G10000 |
| 5732549 | 0.179 | 0.03573814 | SH3 domain protein | AFUA_1G13610 |
| 5735341 | 0.166 | 0.029857932 | purine-cytosine permease | AFUA_2G09860 |
| 5743818 | 0.165 | 0.014768898 | cell cycle control protein Cwf14/Bud31 | AFUA_5G05610 |
| 5745210 | 0.163 | 0.037491668 | conserved hypothetical protein | AFUA_5G12035 |
| 5741172 | 0.161 | 0.008989843 | Glutamate/Leucine/Phenylalanine/Valine dehydrogenase | AFUA_4G06620 |
| 5748265 | 0.136 | 0.045500435 | H+/nucleoside cotransporter | AFUA_6G13190 |
| 5738847 | 0.134 | 0.038979594 | AMMECR1 family protein | AFUA_3G09390 |
| 5742705 | 0.131 | 0.022812879 | MFS transporter, putative | AFUA_4G14230 |
| 5736911 | 0.121 | 0.03631862 | hypothetical protein | AFUA_2G17305 |
| 5730436 | 0.116 | 0.010113819 | MFS sugar transporter, putative | AFUA_1G02530 |
| 5743018 | 0.101 | 0.01797061 | conserved hypothetical protein | AFUA_5G00870 |
| 5731954 | 0.093 | 0.016245933 | ammonium transporter (Mep2), putative | AFUA_1G10930 |
| 5732457 | 0.076 | 0.013750276 | uridine permease Fui1, putative | AFUA_1G13210 |
| 5730314 | 0.075 | 0.006092317 | conserved hypothetical protein | AFUA_1G01960 |
| 5733093 | 0.065 | 0.01788488 | conserved hypothetical protein | AFUA_1G16070 |
| 5733419 | 0.061 | 0.024219496 | high affinity nitrate transporter NrtB | AFUA_1G17470 |
| 5743196 | 0.051 | 0.009742286 | DUF1445 domain protein | AFUA_5G01690 |
| 5733915 | 0.050 | 6.40368E-05 | conserved hypothetical protein | AFUA_2G02000 |
| 5746300 | 0.035 | 0.00791494 | oligopeptide transporter, putative | AFUA_6G03140 |
| 5741273 | 0.023 | 0.013142448 | ornithine carbamoyltransferase | AFUA_4G07190 |
| 5736469 | 0.015 | 0.005435941 | small oligopeptide transporter, OPT family | AFUA_2G15240 |
